# Subcortical Ischemic Vascular Cognitive Impairment: Insights from Reaction Time Measures

**DOI:** 10.3233/JAD-190889

**Published:** 2019-11-26

**Authors:** Emma Richards, Antony Bayer, Jeremy J. Tree, Claire Hanley, Jade E. Norris, Andrea Tales

**Affiliations:** a Centre for Innovative Ageing, Swansea University, Swansea, UK; b Department of Medicine, Cardiff University, Cardiff, UK; c Department of Psychology, Swansea University, Swansea, UK; d Department of Psychology, Bath University, Bath, UK

**Keywords:** Intra-individual variability, methodology, reaction time, subcortical ischemic vascular cognitive impairment

## Abstract

In this study, reaction time (RT), intraindividual variability (IIV), and errors, and the effects of practice and processing load upon such function, were compared in patients with subcortical ischemic vascular cognitive impairment (SIVCI) [*n* = 27] and cognitively healthy older adults (CH) [*n* = 26]. Compared to CH aging, SIVCI was characterized by a profile of significantly slowed RT, raised IIV, and higher error levels, particularly in the presence of distracting stimuli, indicating that the integrity and/or accessibility of the additional functions required to support high processing load, serial search strategies, are reduced in SIVCI. Furthermore, although practice speeded RT in SIVCI, unlike CH, practice did not lead to an improvement in IIV. This indicates that improvement in RT in SIVCI can in fact mask an abnormally high degree of IIV. Because IIV appears more related to disease, function, and health than RT, its status and potential for change may represent a particularly meaningful, and relevant, disease characteristic of SIVCI. Finally, a high level of within-group variation in the above measures was another characteristic of SIVCI, with such processing heterogeneity in patients with ostensibly the same diagnosis, possibly related to individual variation in pathological load. Detailed measurement of RT, IIV, errors, and practice effects therefore reveal a degree of functional impairment in brain processing not apparent by measuring RT in isolation.

## INTRODUCTION

Cerebral small vessel disease in older adulthood, typically appearing as periventricular white matter lesions or leukoaraiosis (LA) [1] on neuroimaging, can result in the development of subcortical ischemic vascular cognitive impairment (SIVCI). This can manifest initially as subjective or subclinical cognitive decline, and then later as minor or major neurocognitive disorder (dementia) [2–10].

Clinical diagnosis and research in early disease, and the ability to identify individuals at greater risk of developing significant cognitive and functional impairment, can be particularly challenging. This is because the onset of SIVCI tends to be insidious as some degree of cerebrovascular disease and LA is common in aging *per se* [8, 11–14]. Moreover, the course of the disease is heterogeneous, with significant individual variation in signs and symptoms [13]. Furthermore, increasing evidence indicates that pathological change in white matter can be ‘silent’, i.e., is not visible (and thus rateable) as hyperintensity on diagnostic neuroimaging [11, 12, 15–17]. It is possible, therefore, that individual pathological change, and its potential impact, may be underestimated. Specifically, white matter changes revealed by neuroimaging do not necessarily relate to cognitive or clinical status and function and the location (not simply the amount) of white matter damage influences cognitive integrity and its specificity [2, 3, 9, 17–20]. Consequently, further information about what functional changes might characterize SIVCI would be of value, especially in terms of helping to understand and explain the basis of some of the signs, symptoms, and behavioral and social challenges associated with SIVCI. Furthermore, the examination of individual differences in such function between patients with ostensibly the same level of disease, can inform a stratified medicine approach. In the present study we therefore examine reaction time (RT) and a series of related measures in SIVCI compared to cognitively healthy older adults (CH).

### Reaction time

There is a robust association between slowed behavioral RT (particularly that related to executive function) and reduced structural and functional integrity of white matter at both regional and global levels [1, 17–19, 21–28]. Predictably, therefore, RT slowing appears to be a significant clinical and research characteristic of SIVCI.

As detrimental changes in white matter are characteristic of vascular cognitive impairment (VCI), one would predict significant RT slowing to characterize VCI, particularly as behavioral RT represents the outcome of extensive network recruitment and processing (for example, in the measurement of executive function-related RT) [3, 5–9, 14, 15, 21, 29–38]. Nevertheless, although routine assessment may include the measurement of executive-function-related RT, there is a lack of consensus regarding which test to use [14, 15, 33, 34, 39]; this is an important issue as the tests will vary with respect to processing loads and possibly therefore their sensitivity to disease presence [41]. Furthermore, whereas research tends to adopt a network approach to RT (where RT is interpreted as the product of distributed neural networks and thus likely to be highly sensitive to neurological impairment) in which related factors such as the intra-individual variability of RT (IIV), error production, and the influence of practice and processing load effects are investigated [21, 22, 41], a common tacit assumption is that only RT is of clinical relevance.

### Intra-individual variability of reaction time

IIV is a behavioral representation of the transient fluctuation of RT over a given number of trials related to various aspects of information processing. These include (but are not limited to) attentional control and lapses, stimulus- and post-perceptual- processes and strategies, the functional and structural integrity of white and grey matter, and the status of distributed neural, and neurobiological networks [26, 27, 42–54]. Although RT and IIV can correlate (i.e., slower RT associated with greater IIV), thus appearing to share common networks, the relationship between them is not always linear. They can dissociate, varying across individuals and age groups and disease and with respect to the number of trials presented [34, 55, 56]. Such evidence indicates that RT and IIV have some degree of independence in terms of underlying processing and networks, which in turn could be differentially affected by aging, disease, and disease progression [33, 55] and individual differences. In an original approach, the relationship between RT and IIV in SIVCI is also examined in this study. Furthermore, IIV appears to be particularly representative of everyday functioning, cognitive status, the risk of falls, injury, health, decline in cognitive function, impending decline, lower functionality, morbidity, and mortality [47, 48, 50, 54, 57–59]. Arguably therefore, IIV may be a more sensitive or meaningful marker of SIVCI than RT alone, and one which may help to improve the functional and clinical characterization of SIVCI.

### Practice effects

In the RT and IIV research domain, multi-trial tests are commonly used to provide additional information about the integrity of complex network control systems, such as processing flexibility, practice effects and error production, the brain’s potential to benefit from short-or long-term training, and learning-related neural modulation and neuroplasticity [42, 46, 53, 54, 56, 60–77]. Such information is not, however, determined alongside RT speed in clinical practice and has not been previously applied to better inform our understanding of SIVCI.

### Study aims

The aim of this study is to use a simple, multi-trial, visual search task to examine RT, IIV, error production, the effect of processing load (specifically induced by the addition of distracting information), and practice effects (comparing the outcome from the first and last ten trials) at group mean level in individuals with SIVCI compared to CH. RT and IIV within the SIVCI group will also be examined in order to determine how individuals with ostensibly the same diagnosis may vary in such performance.

## METHODOLOGY

### Ethical approval

The study protocol was approved by the NHS Health and Research Authority Wales Research Ethics Committee 6, and Research and Development, Cardiff and Vale NHS Trust. Written informed consent was obtained from all participants.

### Participants with subcortical ischemic vascular cognitive impairment

Patients with SIVCI (diagnosed according to Skrobot et al. [10]) were recruited on an incident patient basis from the Memory Clinic at University Hospital Llandough, Wales, UK. An invitation letter which included a participant information sheet, researcher contact details, an opt-in form and pre-paid envelope, was sent to all individuals who expressed an interest in participation. For the SIVCI patient group (*n* = 27), individuals were diagnosed with minor or major neurocognitive disorder associated with lacunar infarcts and ischemic white matter lesions as the main type of brain lesions, located predominantly subcortically [10, 78]. Diagnosis was made after comprehensive assessment according to normal clinical practice. This included neuroimaging (normally CT scans, or MRI scans if requested), detailed clinical history, routine laboratory tests, and a battery of neuropsychological tests assessing executive function, attention, memory, language, visuospatial function (Addenbrooke’s Cognitive Examination III [79]) and the Montreal Cognitive Assessment (MoCA) [80], premorbid ability (National Adult Reading Test (NART) [81], and mood (Hospital Anxiety and Depression Scale (HADS) [82]). Inclusion criteria included capacity to provide informed consent, mild to moderate cognitive impairment (MoCA score between 12 and 25 and/or ACE-III score between 50 and 90), normal or corrected-to-normal vision and hearing, and physical ability to perform the research tasks. Exclusion criteria included: other significant contributory cause of cognitive impairment (e.g., clinically significant neurological, psychiatric, psychological, or medical conditions), use of psychoactive drugs, substance or alcohol dependency, and motor/manual dexterity problems. The CT and MRI scans examined as part of this study were those performed for diagnostic purposes and were examined with respect to the presence of subcortical and cortical infarcts and LA, mass lesion, focal atrophy or other significant pathology. The extent of periventricular LA was assessed using the age-related white matter changes rating scale (ARWMC) [83], with 0 = no lesions; 1 = focal lesions, 2 = beginning confluence of lesions, 3 = diffuse involvement of the entire region. Assessment was undertaken by two experienced professionals in the field (AB and AT) who independently rated each scan, yielding a 93% (25 out of the 27 scans) consensus rate. The remaining two scores were agreed by consensus.

### Cognitively healthy older adult controls

The cognitively healthy older adult control group (CH) (*n* = 26) were recruited from relatives of patients attending the Llandough Memory Clinic and participating in this study, and from research volunteers from the Centre for Innovative Ageing (CIA), the Centre for Ageing and Dementia Research (CADR), and the older adult research volunteer database at Swansea University. Inclusion criteria included capacity to provide informed consent, MoCA score of > 25, normal or corrected-to-normal vision and hearing, and physical ability to perform the research tasks. Exclusion criteria included self-reported cognitive change or impairment, or past visits to their general practitioner or memory services regarding such concerns, significant neurological, psychiatric, or medical conditions, psychoactive drug use, and current or history of substance or alcohol dependency. The use of prescribed and non-prescribed medication was recorded but not controlled. The CH group was age-matched as closely as possible to the SIVCI group. Neuroimaging was not available for the control group.

### Demographics

Table 1 details the demographics for the CH older adults and the SIVCI patient group.

**Table 1:** Demographic details for the cognitively healthy (CH) older adults and the SIVCI patient group (standard deviation in parenthesis)

	CH (*n* = 26)	SIVCI (*n* = 27)
Age: mean (SD) [y]	76.219 (5.51)	78.11 (6.14)
Age range [y]	70–86	68–91
Gender (%)	26.9% Male	51.9% Male
FT education: mean (SD) [y]	15.8 (4.0)	12.3 (2.7)
Educational range [y]	10–22	8–21
MoCA score; mean (SD)	28.1 (1.4)	19.9 (3.3)
HADS score – anxiety: mean (SD)	5.7 (3.8)	6.08 (3.68)
HADS score – depression: mean (SD)	2.9 (2.86)	4.29 (3.43)

### The Visual Search Test

#### Rationale

We employed a computer-based multi-trial visual search test (e.g., [84]) to facilitate the concurrent determination of RT, IIV, error production, and practice effects *per se* and any interactions between them. We also examined how task processing load; namely the detrimental influence of distracting information, can influence such measures.

#### Task description

**Fig.1:**
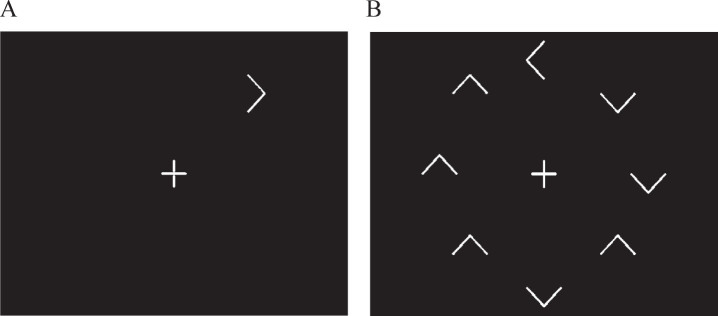
Representation of the target alone (distracter absent) and target with distractors (distracter present) visual search conditions.

In the visual search test, the time taken to respond to whether a target (a white arrow) was pointing to the left or right of the screen, was determined for each participant when it appeared both in isolation (Fig. 1A) and surrounded by similar but irrelevant distracting stimuli (Fig. 1B), namely seven other white arrows pointing up or down. Surrounding the target with distracting information significantly reduces the saliency of the target, and thus its ease of detection, thereby invoking a serial search strategy in order to discover the target. Such a strategy requires the recruitment of additional functions and processing resources, any, or all of which may be differentially influenced by SIVCI compared to CH, thus potentially providing additional behavioral measures characteristic of SIVCI.

The stimuli were generated on a Toshiba Satellite Pro A50-C-1GC laptop with a 15-inch screen. The white target and distracters were displayed upon a black screen at a viewing distance of 57 cm. A clock face configuration of stimulus presentation ensured counterbalanced stimulus presentation in order to account for potential differences in processing between the upper, lower, and lateral visual fields. There were two visual search conditions. In the distracter absent (DA) condition, the target was presented in isolation (Fig. 1A). In the distracter present (DP) condition, the same target was presented surrounded by seven irrelevant but distracting arrows pointing either up or down (Fig. 1B). Each target or distracter element appeared radially and equidistant from the intersection of the lines forming the fixation cross and when all eight appeared, were equally spaced. For each trial, the central fixation cross appeared on screen for 1000 ms prior to the appearance of the target and remained on screen for the duration of the trial. The stimuli remained on the screen until the participant responded, after which the next trial appeared. A total of 64 trials were presented, 32 for the DA, and 32 for the DP conditions, with the target appearing eight times at each of the possible ‘clock-face’ locations. Target response was by means of a three-button row stimulus box attached to the laptop via USB cable; pressing the left button if the target was pointing left and the right button if the target was pointing right (the middle button being redundant for this task). Participants were instructed to fixate on the center cross at the beginning of each trial and to respond as quickly but as accurately as possible. After instruction, all participants were asked to describe what they had to do for the task in order to ensure understanding and were then required to perform a practice block of ten trials. The ability of the participants to fixate on the cross at the beginning of each trial continued to be checked throughout the procedure by researcher observation. Performance feedback was not given.

### Data analysis

Based on consensus in this field (see [33, 53, 54]), for each participant, for each condition, a 150 ms minimum cut off point was applied in order to exclude anticipatory responses, i.e., those that are faster than the time needed for decision and motor action components. Any such responses were removed from data analysis and recorded as errors. Data resulting from response error (pressing the wrong button), obvious lapses of attention or other unintentional interruption (leading to extreme outliers) were also removed from each individual’s data and also recorded as errors. The median RT and IQR (IIV) data for each participant were then entered into group analysis. The RT data were not normally-distributed, and log transformed data also failed to conform to normality of distribution. Thus, as in Phillips et al. [33], the data were analyzed using analysis of variance (ANOVA), as the *F*-test is a valid statistical procedure to control for Type 1 error under non-normality conditions [85]. We ensured a robust statistical approach by also subjecting the data to non-parametric analysis, but as the outcome of such analysis did not differ from that using ANOVA (or indeed the log transformed data), we report here only the parametric analysis in line with common practice [33]. To aid study outcome comparison and the meaningfulness of our results, we also report Cohen’s effects sizes and 95% confidence intervals.

## RESULTS

### Demographics

Independent samples *t*-test analysis revealed no significant differences in mean age, anxiety, or depression scores between the CH and SIVCI groups (all *p-*values > 0.05), whereas mean educational level was significantly lower for the SIVCI compared to the CH group [t (44.72) = 3.7, *p* = 0.001, Cohen’s *d* = 1.005, (equal variances not assumed), 95% CI (1.5, 5.21)].

### Visual search: All trial analysis

Mean RT, IIV, and error values based on the median individual scores (standard deviation in parenthesis) for the CH and SIVCI groups are shown in Table 2.

**Table 2:** Mean RT, IIV, and error values based on the median individual scores (standard deviation in parenthesis) for the CH and SIVCI groups

	RT (ms)		IIV (ms)		Errors	
	CH	SIVCI	CH	SIVCI	CH	SIVCI
Distractor absent (DA)	734.25 (157.58)	1133.82 (671.31)	222.81 (103.7)	561.6 (609.5)	0.023 (0.04)	0.052 (0.06)
Distractor present (DP)	1681.44 (309.27)	3232.04 (2290.06)	973.7 (295.5)	2275.8 (1805.1)	0.025 (0.06)	0.085 (0.1)

### RT

Mixed design ANOVA on group (CH, SIVCI; between group factor), and search condition (DA, DP; within group factor), revealed a significant main effect of group [F (1,51) = 12.73, *p* = 0.01, *η*p^2^ = 0.20] in which overall RT was significantly slower for the SIVCI compared to the CH group, with further independent *t* test analysis revealing this effect for both the DA [t (28.96) = –3.01, *p* = 0.005, *d* = –0.96 (equal variances not assumed) (95% CI (–671.27, –127.87)] and the DP [t (26.98) = –3.49, *p* < 0.002, *d* = –1.19 (equal variances not assumed), 95% CI (–2463.43, –637.76)] conditions. There was also a significant main effect of target condition [F (1,51) = 62.38, *p* < 0.01, *η*p^2^ = 0.55], whereby RT was significantly slower for the DP compared to the DA condition for both the CH [t (25) = –21.35, *p* < 0.001, *d* = –5.34) 95% CI (–1038.57, –855.82)] and the SIVCI [t (26) = –5.58, *p* < 0.001, *d* = –1.61) 95% CI (–2870.72, –1325.72)] groups; and a significant target by group interaction [F (1,51) = 8.91, *p* = 0.01, *η*p^2^ = 0.15] in which the difference in RT between the DP and the DA conditions was significantly greater for the SIVCI compared to the CH group [t (26.72) = –3.04, *p* = 0.05, *d* = –1.06 (equal variances were not assumed), 95% CI (–1927.87, –374.19)]. It is possible that the significant difference in RT between the two groups could be explained by the significantly higher educational level of the CH group. However, further univariate ANOVA with educational level as covariate revealed that the significant difference in RT between the two groups remained after controlling for educational level [F (1, 50) = 5.49, *p* = 0.023].

For the DA condition, RT was not significantly correlated with educational level for either the CH or SIVCI groups (all *p*-values > 0.05). For the DP condition, RT was significantly negatively correlated with educational level for the CH group, with lower levels of education associated with slower RT (*r* = –0.54, *p* = 0.005), whereas RT was not significantly correlated with educational level for the SIVCI group (*p* > 0.05).

For both the CH and SIVCI groups, further independent *t* test analysis revealed that RT did not vary significantly with respect to gender for the DA condition. For the DP condition, although RT did not vary significantly with respect to gender for the SIVCI group (*p* > 0.05), RT was significantly slower for females [t (23.29) = –3.69, *p* = 0.001 (equal variances not assumed)] in the CH group.

### IIV analysis

Mixed design ANOVA on group (CH and SIVCI) and target (DA, DP) revealed a significant main effect of group [F (1, 51) = 14.44, *p* < 0.01, *η*p^2^ = 0.22], namely, a greater level of IIV for the SIVCI compared to CH group, with further independent *t* test analysis revealing this effect for both the DA [t (27.5) = –2.85, *p* = 0.008, *d* = –0.95, 95% CI (–582.79, –94.70)], and DP [t (27.5) = –3.70, *p* < 0.001, *d* = –1.24 (equal variances not assumed), 95% CI (–2024.12, –579.98)] conditions. There was also a significant main effect of target [F (1,51) = 60.66, *p* < 0.01, *η*p^2^ = 0.54], in which IIV was significantly greater when the target was surrounded by distracting information, with further independent *t* test analysis occurred for both the CH [t (25.0) = –13.33, *p* < 0.001, *d* = 3.08, 95% CI (–866.92, –634.92)] and the SIVCI [t (26.0) = –5.61, *p* < 0.001, *d* = –1.42, 95% CI (–2342.65, –1085.79)] groups. Finally, there was a significant target by group interaction [F(1,51) = 9.26, *p* < 0.05, *η*p^2^ = 0.15], in which the distracter effect, namely the influence of the distractors upon IIV was significantly greater for the SIVCI compared to the CH group [t (27.77) = –3.10,=*p*<0.005, *d* = –1.03 (equal variances not assumed) 95% CI (–1600.33, –326.26)]. Further univariate ANOVA analysis with educational level as covariate revealed that the significant group differences in IIV remained after controlling for educational level [F (1,50) = 6.04, *p* = 0.017]. For both target conditions, for both groups, further independent *t* test analysis revealed that IIV did not vary significantly with respect to gender (all *p*-values > 0.05). For the DA condition, for both groups, IIV was not significantly correlated with educational level (*p* > 0.05). For the DP condition, IIV was significantly negatively correlated with educational level (*r* = –0.393, *p* = 0.047) for the CH group, with lower levels of education associated with greater levels of IIV, but not for the SIVCI group (*p* > 0.05).

### The relationship between RT and IIV_RT_

For the DA condition, RT and IIV were significantly correlated for the SIVCI group (*r* = 0.85, *p* < 0.001) with higher levels of IIV associated with slower RTs, but not for the CH group (*p* > 0.05). For the DP condition, RT and IIV were significantly correlated (*r* = 0.52, *p* = 0.006) and (*r* = 0.81, *p* < 0.001) for both the CH and SIVCI groups, respectively, with higher levels of IIV associated with slower RTs.

### Error analysis

Although the average number of errors was small for both groups, independent *t* test analysis revealed that the SIVCI group made significantly more errors than the CH group, for both the DA [t (43.1) = –2.2, *p* = 0.04, *d* = 0.59, 95% CI (–0.06, –0.002)] and DP [t (41.72) = –2.7, *p* = 0.01, *d* = 0.74, 95% (equal variances not assumed) 95% CI (–0.1, –0.01)] conditions. Further independent *t* test analysis revealed that although the addition of distracters did not significantly change the number of errors for the CH group (*p* > 0.05), they significantly increased the number of errors for the SIVCI group [t (26) = –2.3, *p* = 0.03, *d* = 0.4, 95% CI (–0.06, –0.003)]; with none of the results varying significantly with respect to gender (all *p*-values > 0.05). For both target conditions, there was no significant correlation between errors and educational level for either the CH or SIVCI group (all *p* values > 0.05).

### Periventricular white matter disease

Results based on the ARWMC [83] in the SIVCI group are shown in Table 3.

**Table 3:** Age-related white matter changes rating scale (ARWMC) [83], in the SIVCI group (mild = 0/1, moderate/severe = 2/3). Standard deviation in parenthesis

	ARWMC rating scale of periventricular white matter disease	Number of participants	Mean RT (sd)	Mean IIV (sd)	Mean number of errors (sd)
Distracter absent (DA)	Mild	15	1223.8 (859.3)	636.1 (739.3)	0.07 (0.07)
	Moderate/severe	12	1021.3 (317.2)	468.4 (406.8)	0.03 (0.05)
Distracter Present (DP)	Mild	15	3129.3 (1792.9)	2176.0 (1653.4)	0.09 (0.09)
	Moderate/severe	12	3360.4 (2876.2)	2400.5 (2047.3)	0.08 (0.11)

For both the DA and DP conditions, there was no significant difference in RT, IIV, or errors between mild and moderate/severe levels of periventricular white matter disease level (all *p*-values > 0.05). Spearman’s correlational analysis also revealed no significant correlation between white matter score and RT, IIV, or errors (all *p*-values > 0.05). Note however that white matter score was significantly correlated with age (*r* = 0.48, *p* = 0.012).

### Practice effects in RT, IIV, and errors

Mean RT and IIV and errors for the first and last ten trials for the CH (*n* = 26) and SIVCI (*n* = 27) groups are shown in Table 4.

**Table 4:** Mean RT and IIV and errors for the first and last ten trials for the CH (*n* = 26) and SIVCI (*n* = 27) groups. Standard deviation in parenthesis

Target condition	Trial	Group	Mean RT (ms)	Mean IIV (ms)	Mean Errors (sd)
Distracter Absent (DA)	First 10	CH	774.92 (179.92)	273.72 (154.00)	0.039 (0.085)
		SIVCI	1212.78 (872.52)	684.2 (863.41)	0.056 (0.080)
	Last 10	CH	739.63 (181.74)	228.96 (148.55)	0.007 (0.027)
		SIVCI	1132.00 (638.33)	550.7 (460.9)	0.044 (0.101)
Distracter Present (DP)	First 10	CH	1814.75 (551.35)	1145.94 (422.73)	0.035 (0.080)
		SIVCI	3474.15 (2329.06)	2540.02 (1805.07)	0.070 (0.11)
	Last 10	CH	1693.33 (411.69)	922.15 (319.97)	0.000 (0.00)
		SIVCI	3030.28 (1605.50)	2269.07 (2144.95)	0.078 (0.125)

#### Reaction time

For the DA condition, there was no significant difference in RT between the first and last 10 trials for both the CH and SIVCI groups [t (25) = 1.69, *p* = 0.104] and [t (26) = 1.2, *p* = 0.24], respectively. For the DP condition, although there was no significant difference in RT between the first and last 10 trials for the CH group [t (25) = 1.1, *p* = 0.3], for the SIVCI group, RT was significantly faster for last compared to the first ten trials [t (26) = 2.1, *p* = 0.05, *d* = 0.2].

#### Intra-individual variability

For the DA condition, there was no significant difference in IIV between the first and last 10 trials, for the either the CH [t (25) = 1.27, *p* = 0.22] or the SIVCI [t (26) = 0.979, *p* = 0.34] groups. For the DP condition, IIV was significantly reduced for the last compared to the first ten trials for the CH [t (25.0) = 2.46, *p* = 0.02. *d* = 0.6] but not for the SIVCI group [t (26.0) = 0.86, *p* = 0.4].

#### Error analysis

For the DA condition, there was no significant difference in errors between the first and last 10 trials for either the CH [t (25) = 1.69, *p* = 0.1] or the SIVCI [t (26) = 0.46, *p* = 0.65] groups. For the DP condition, the number of errors was significantly reduced in the last compared to the first ten trials for the CH [t (25) = 2.21, *p* = 0.4, 95% CI (0.002, 0.07)], but not for the SIVCI (*p* > 0.05) group.

### Level of white matter disease

For both target conditions, within the SIVCI group, there was no significant difference in RT, IIV, and errors between the mild versus moderate/severe levels of periventricular white matter disease. Furthermore, RT, IIV, and errors were not significantly correlated with level of white matter disease (all *p-*values > 0.05).

## DISCUSSION

The aim of this study was to examine RT, IIV, errors, practice effects, and processing load in SIVCI compared to CH aging using a computer-based, multi-trial, visual search paradigm.

### Summary of main findings

Compared to CH aging, SIVCI has a profile of significantly slowed RT, raised IIV and error levels, a disproportionately greater detrimental response to high processing load conditions (namely the presence of distracting environmental information), a lack of improvement in IIV with practice, and a high degree of individual differences in the performance of all these functions.

### Reaction time and intraindividual variability

Target RT was significantly slower, and IIV significantly greater, in SIVCI irrespective of whether the target was surrounded by distracting information or not. However, the detrimental effect of adding distracters, namely RT slowing and increased IIV, was disproportionately greater for the SIVCI compared to the CH group. This indicates that the integrity and/or accessibility [40] of the additional functions required to support the high processing load, serial search strategy, invoked when distracting information surrounds the target, are reduced in SIVCI; a functional decline likely to significantly disrupt everyday life [86, 87]. The examination of such aspects of information processing therefore not only helps to characterize SIVCI, but also indicates the type of environment likely to induce processing failure.

Although there is some degree of variation in RT within the CH group, it is apparent to a much greater degree within the SIVCI group (see Table 2). This finding is in accord with previous evidence indicative of heterogeneity in other aspects of cognitive function in SIVCI (e.g., [13]). Arguably, such processing heterogeneity in patients with ostensibly the same diagnosis, may be related to individual variation in pathological load. Although there was some evidence in support of this suggestion, namely that patients with moderate/severe levels of periventricular LA showed slower RT and higher IIV than those patients with mild levels for the DP task, these differences failed to reach statistical significance and, in response to the DA condition, performance was worse (but again not significantly so) for the mild subgroup. It is likely that the lack of significance is a result of the low numbers of participants in each of the SIVCI subgroups (mild *n* = 15, versus moderate/severe, *n* = 12); nevertheless, it is also possible that the level of CT- or MRI-visible periventricular white matter change alone does not fully explain the highly significant slowing and raised IIV in SIVCI compared to CH, which may be the result also of the impact of ‘silent’ white matter disease and/or other disease-related changes in SIVCI. Further research is necessary in order to determine whether RT and IIV and associated measures may also be of use as adjuncts to neuroimaging in the estimation of disease burden.

Examining within-group heterogeneity (standard deviation) in SIVCI also revealed the presence of certain individuals for whom performance levels are worse than expected for group mean levels. As some evidence from the study of mild cognitive impairment [88, 89] indicates that individuals with slower RT or raised IIV are at greater risk of disease progression, one can speculate that SIVCI patients with particularly slow RT or high IIV, are those most at risk of disease progression, or are, in fact, at a later stage of disease than that indicated by neuropsychological and other test results. Moreover, although both RT and IIV appear similarly able to differentiate SIVCI from CH, the greater association of IIV with health and functional status [47, 48, 50, 54, 57–59], indicates that IIV may be a more sensitive or meaningful characteristic of VCI than RT alone, and should therefore be measured alongside RT in clinical practice. Again, further research is required to appropriately investigate such speculation.

In the easier, less resource-demanding DA condition, RT and IIV were not significantly correlated for the CH group; an indication of dissociability between RT and IIV [55, 56]. In contrast, for the SIVCI group, RT and IIV were significantly correlated, with higher levels of IIV associated with slower RTs. In the harder, or higher processing load, DP condition, RT and IIV were significantly correlated for both the CH and the SIVCI groups. This pattern of results indicates that in CH aging, RT and IIV are significantly correlated only in response to difficult or high resource-demanding processing conditions, whereas for the SIVCI group, they are correlated for low, as well as high, resource demanding tasks. Correlation between RT and IIV in response to simple, low processing load tests may therefore be a further sign of disease [33, 54–56, 60–68]. Further research is required in order to replicate such results and to determine their clinical relevance.

A characteristic of the SIVCI patients in this study was their significantly lower educational level [90–92]. Although the group difference in RT and IIV remained after controlling for educational level, educational level was significantly negatively correlated with both RT and IIV for the CH group, but only under DP conditions. A processing advantage not apparently accessible to those with higher levels of education in the SIVCI group possibly as the higher level of education in the SIVCI group was less than that for the CH group.

### Errors

Although errors were low for both groups, the SIVCI group made significantly more than the CH group under both the DA and DP conditions, and only for the SIVCI group did the addition of distracters significantly increase the number of errors compared to the DA condition, results that remained after accounting for educational level. This further emphasizes the detrimental effect distracting stimuli have upon information processing in SIVCI. Despite the lack of a significant correlation between the level of visible subcortical periventricular white matter lesions and the number of errors, errors are also associated with the functional integrity of complex processing networks [56, 60–62, 64–66, 93], their increased prevalence in SIVCI, especially in response to conditions with high processing demands, are also indicative of breakdown in processing networks, and thus potential for disruption to normal behavior.

### Practice effects

For the DA condition, RT, IIV, and the number or errors did not differ significantly between the first and last ten trials, for either the CH or the SIVCI group. Practice did not therefore significantly improve performance in either group; a stability possibly reflecting the relatively low processing level demands of this condition, and that for both groups, processing efficiency was already at its maximum possible level at the beginning of the task and thus could not be improved by practice.

For the more resource-demanding DP condition, practice resulted in a significant reduction in RT, but no significant change in IIV or errors for the SIVCI group, and for the CH group no significant change in RT or errors, but a significant reduction in the degree of IIV. Although this provides some evidence of the ability of individuals with SIVCI to improve RT performance with practice, the effect size was relatively small (0.2) and RT did not approach that typical of CH aging. This may reflect the fact that the SIVCI group were slower at the beginning of the task and thus had a greater ‘scope’ for improvement than the CH group, and that the CH group may have been performing at maximum from the beginning of the test.

Although the underlying cause for this pattern of results remains to be determined, they indicate that improvement in RT can in fact mask an abnormally high degree of IIV. Because IIV appears more related to disease, function, and health than RT [47, 48, 50, 54, 57–59], its status may therefore (with further investigation) represent a more meaningful, relevant disease characteristic than RT in SIVCI.

### Conclusion

Detailed measurement of RT, IIV, errors, and practice effects can show a range of functional impairment in brain processing not apparent by measuring RT in isolation. Although such measures help to explain the basis for some of the behavioral signs and symptoms of SIVCI, further larger scale studies are required to determine whether such measures represent clinically useful adjuncts to the use of diagnostic neuropsychological tests and neuroimaging-visible white matter lesions, in the diagnosis of SIVCI and disease level.

### Study strengths and limitations

The strengths of this study include the fact that the participant numbers recruited and tested in this study reflect those typically used in such research investigation of RT and IIV in aging and clinical populations [88, 89] and have resulted in high effect sizes indicative, with further development, of potential clinical utility in the measurement of RT, IIV, and errors and the search paradigm. A further strength was the ability to measure such a wide range of functions using just one, simple to understand and easy to perform, test. Potential limitations include the lack of patient numbers required to appropriately investigate any relationship between the level of periventricular LA (mild versus moderate/severe) and behavioral RT and IIV, the lack of inclusion of a wider range of trial numbers and of task processing resource requirements, the absence of neuroimaging data for the CH group, and the use of only limited, clinical scans in the judgement of white matter lesion loads within the SIVCI group. Furthermore, for the majority of the participants with SIVCI, only a CT rather than MRI scan was available, and although CT has more limitations than MRI with respect to the visualization of white matter lesions, the preference for CT reflects national health service (NHS) practice. In addition, we were unable to perform CT/MRI scans for the cognitively healthy older adult control group, with the lack of DTI scans for either group precluding the ability to examine the relationship between global measures of white matter integrity and RT and IIV.

In the future, we suggest a neuroimaging study with longitudinal assessment (follow up at six-month intervals) including voxel-based morphometry to assess grey matter volume change, diffusion-weighted imaging for white matter integrity (particularly analysis of radial diffusivity as a marker of demyelination) as well as performing executive function tasks during fMRI, and potentially resting state as well, in order to obtain evidence of a relationship between behavioral RT and IIV performance, and structural and functional change over time. We also plan to further examine RT and IIV with respect to variation in the number of trials performed, the boundaries for splitting trial numbers, individual asymptote levels, strategies, and adaptive testing [64, 67, 94].
